# Books and Pamphlets Received

**Published:** 1886-09-20

**Authors:** 


### BOOKS AND PAMPHLETS RECEIVED.

*The Facts and Mysteries of Spiritualism;* with a Sequel. By T. W. Hartley & Co., 420 Franklin street, Philadelphia, Pa. Price, $1.50.

This book gives a detailed account of the writer's experience in the investigation of the subject of Spiritualism. Few men have been able to deal experimentally with spiritualism and come out of it safe and unscathed. How much of the facts and incidents related by Mr. Hartwell are due to ultra mundane influences and how much to an exalted and oversensitive imagination it is i r.pos sible to know. The human mind, or the *electro negative* or passive state, is for the time wholly subject to extraneous influences. The brain is dual organ, and, in certain conditions, it would seem that one side of the brain converses by thought with the other side. This may be called a waking dream. The will, or the link which in ordinary states ties the two together as one, is here inactive or in abeyance, and the two sides tossed and unguided by the will, which may be called the co-ordinating and balancing power, act separately, yet each being conscious of the thoughts and emotions of the other.

That spirits or minds outside of the body may not, under these circumstances, enter into the human brain and become a party to the mental actions which are going on, we are not prepared to controvert. We are not disposed to deny and ridicule everything which we cannot understand, nor to assume that the thousands of intelligent persons who proclaim the reality of Spirit Phenomena are *all* deluded and crazy. Where there is so much smoke there must be a little fire. With thess views, and with facts in the Scriptures which proclaim the existence, in ancient times, of obussions by wicked spirits, we will not deny the truth of all such pheoomena; nor say that in this day such things cannot exist. We will state, however, our opinion that the state of the brain above described is not a normal philosophical condition, and may, by repetition, become dangerous. The party who allows himself to be frequently placed in such state is liable to lose the full control of his mind, and become a subject of the electro-negative state as a permanent condition.

Mr. Hartwell's book is well written, and the facts of his strange experience are doubtless honestly and faithfully presented, and will well repay perusal, even by those who reject the Spirit theory, as illustrating the varied mysterious phases to which the human mind is susceptible.

*The Genuine Works of Hypocrates.* Translated from the Greek, by Francis Adams, LL. D., Surgeon. New York: William Wood & Co.

Those of our profession who lay claims to literary attainments; those to whom the name of Hypocrates is familiar only through a knowledge of his great deeds; those who are fond of studying and watching the evolutions of medical lore from the remotest antiquity to present date, could not de better than to devote a few hours in the reading of this admirable work. Too much credit cannot be accorded to the translator for the fidelity and accuracy with which he laid bare the theories, plans, methods, and treatment of this great mind of Antiquity, and for the elucidation of many nebulous points in his philosophical and medical treatises. This work commends itself to the scholar, and especially to the intelligent medical reader.

